# Optimization of fermentation conditions for an *Escherichia coli* strain engineered using the response surface method to produce a novel therapeutic DNA vaccine for rheumatoid arthritis

**DOI:** 10.1186/s13036-018-0110-y

**Published:** 2018-10-10

**Authors:** Juan Long, Xiao Zhao, Fei Liang, Nan Liu, Yuying Sun, Yongzhi Xi

**Affiliations:** Department of Immunology and National Center for Biomedicine Analysis, Beijing 307 Hospital, No.8, Dongda Ave, Fengtai District, Beijing, 100071 People’s Republic of China

**Keywords:** Therapeutic DNA vaccine, Chicken type II procollagen, Engineered *Escherichia coli*, Optimizing fermentation conditions, Rheumatoid arthritis

## Abstract

**Background:**

Fermentation condition optimization and nutrients screening are of equal importance for efficient production of plasmid DNA vaccines. This directly affects the downstream purification and final quality and yield of plasmid DNA vaccines. The present study aimed to optimize the fermentation conditions for high-throughput production of therapeutic DNA vaccine pcDNA-CCOL2A1 by engineered *Escherichia coli* DH5α, using the response surface method (RSM).

**Results:**

We hypothesized that optimized fermentation conditions significantly increase the yield of pcDNA-CCOL2A1 therapeutic DNA vaccine, a novel DNA vaccine for treating rheumatoid arthritis (RA). Single-factor analysis was performed to evaluate the optimal basal culture medium from LB, 2 × YT, TB, M9 (Glycerol) and M9 (Glucose), respectively. Thereafter, the Plackett-Burman design (PBD) was used to ascertain the three most significant factors affecting the vaccine yields, followed by the paths of steepest ascent to move to the nearest region of maximum response. Initial screening through the PBD revealed that the most key factors were peptone, mannitol, and inoculum concentration. Subsequent use of RSM was further optimized for the production of therapeutic DNA vaccine pcDNA-CCOL2A1 through Box-Behnken design (BBD). The final optimized fermentation conditions were as follows: peptone, 25.86 g/L; mannitol, 8.08 g/L; inoculum concentration, OD = 0.36. Using this statistical experimental design, the yield of therapeutic DNA vaccine pcDNA-CCOL2A1 markedly increased from 223.37 mg/L to339.32 mg/L under optimal conditions, and a 51.9% increase was observed compared with the original medium.

**Conclusions:**

The present results provide a basis for further production of high-quality and high-yield therapeutic DNA vaccine pcDNA-CCOL2A1 in pilot-scale and even industrial-scale.

## Background

Therapeutic DNA vaccines, especially antigen-specific tolerizing DNA vaccines, as novel therapeutic strategies for rheumatoid arthritis (RA), have displayed marked advantages compared with current therapies including disease-modifying antirheumatic drugs (DMARDs), cytotoxic agents, cytokine antagonists or monoclonal antibodies, tofacitinib, glucocorticoids, etc. [1–3]. These therapies generally control RA disease activity by either suppressing overall immune function or partially neutralizing individual cytokines or partially antagonizing individual cytokine receptors; however, they rarely modulate immune cell populations, except for methotrexate (MTX) [4–8]. Furthermore, they not only cannot cure the disease but also inadequately discontinue disease progression, especially for the invasive destruction of articular cartilage and bone. In particular, they can also increase the potential risk of severe infections and malignancy [4, 5].

Unlike current aforementioned therapies, antigen-specific tolerizing DNA vaccine pcDNA-CCOL2A1 encoding chicken type II collagen exerts its therapeutic effects through specific immune modulation, especially for inducing potent immune tolerance against RA. More precise mechanisms of action include increase in the contents of CD4^+^CD25^+^ T regulatory cells, reductions in the specific proliferative response of T lymphocytes to CII, and induction of a shift from Th1 to Th2 cells, accompanied by down-regulation of Th1-cytokine TNF-α and up-regulation of both Th2-cytokine IL10 and Th3-cytokine TGF-β. Moreover, we previously reported that DNA vaccine pcDNA-CCOL2A1 displayed efficacy comparable to those of the current “gold standard” therapy, methotrexate (MTX), in the established collagen-induced arthritis (CIA) rat model. And it is safe and well-tolerated without any abnormal clinical signs and adverse effects on normal physiological function [9–12], suggesting that this vaccine has a high drugability.

Successful establishment of a three-tier cell bank with high stability and identification of a high-yield *Escherichia coli* DH5α strain to produce therapeutic DNA vaccine pcDNA-CCOL2A1 would yield a sound theoretical and material basis for further pilot-scale tests and even industrial-scale production of this vaccine [13]. Furthermore, fermentation conditions including growth conditions, culture types, and culture medium composition influence the yield and productivity of plasmid DNA vaccines, by directly influencing the downstream purification, quality and yield of plasmid DNA vaccines [14–16]. Since many factors existed in the fermentation condition, a great number of experiments should be simultaneously conducted, and the possible interactions between these factors would be studied. So a reasonable analytical methods will lead to lower reagent consumption and considerably less laboratory work. Conventional single dimensional research gives unreliable results, inaccurate conclusion. Orthogonal testing optimum design can reduce the number of combinations, and obtain optimal factor levels. However, it cannot give a regression equation for the whole parameter tested. By contrast, the combination of Plackett-Burman design (PBD) with common optimization methodology Box-Behnken design-response surface method (BBD-RSM) can collectively eliminate these limitations and are powerful and useful in determining the key factors rapidly from a multivariable system [17]. PBD provides indication and tendency regarding the necessity of each variables in relatively few experiments, the following Box-Behnken design (BBD) provides a large amount of information and the interaction of the independent variables on the response by a small number of experiment [18–21]. The data from BBD subject to a second-order multiple regression equation showing the dependence of the response (i.e. the plasmid yield) on independent variables (i.e. the concentration of the separate components of the nutrient medium or fermentation parameters), and even give predictive results of responses and the possible levels of related independent variables. The equation of the model can clearly present the effects for binary combinations of the independent variables.

Essentially, plasmid DNA vaccine production is aimed at increasing yield and productivity and decreasing manufacturing cost. Hence, we hypothesize that optimization of fermentation conditions significantly increases the yield and productivity of therapeutic DNA vaccine pcDNA*-*CCOL2A1. The present study aimed to determine the effect of optimized fermentation conditionsof the engineered *Escherichia coli* DH5α on the yield of therapeutic DNA vaccine pcDNA-CCOL2A1. To our knowledge, this is the first study to systemically optimize fermentation conditions of the engineered *Escherichia coli* DH5α for producing therapeutic DNA vaccine pcDNA-CCOL2A1 through a combination of the commonly used PBD with common optimization methodology BBD-RSM.

## Results

### Single-factor analysis of basal culture medium revealed optimal carbon and nitrogen sources for producing therapeutic DNA vaccine pcDNA-CCOL2A1

**Fig. 1:**
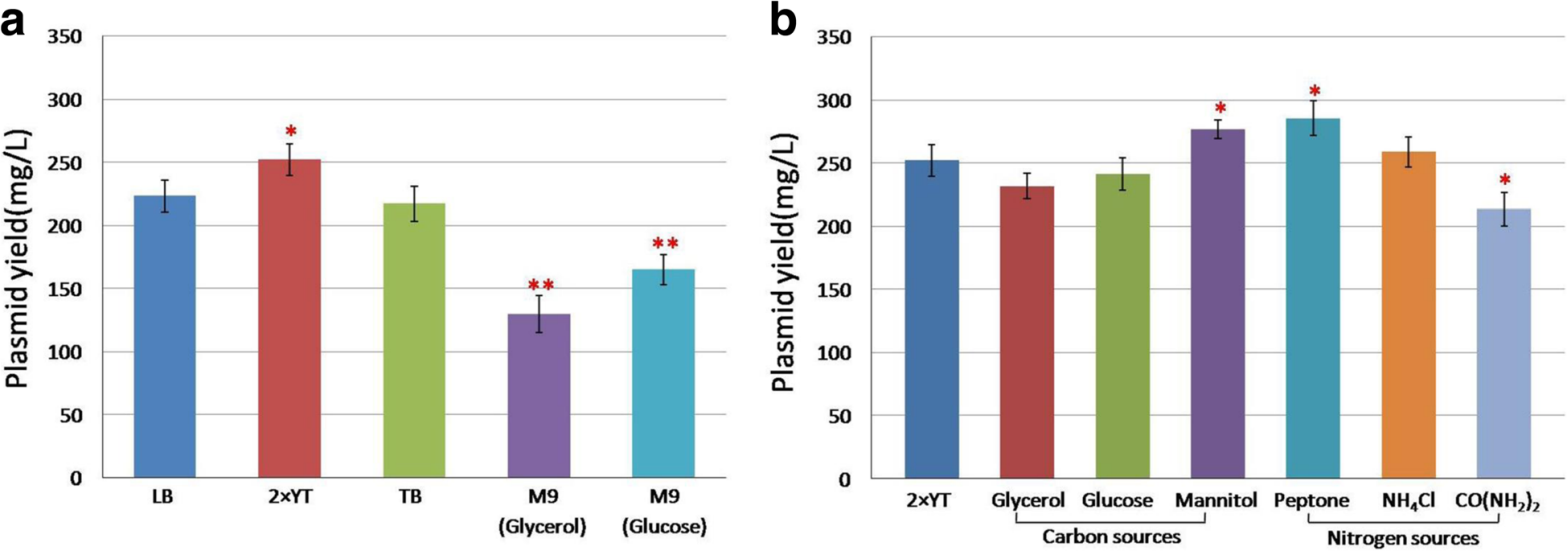
**a**, **b** Single-factor analysis of basal culture medium revealed optimal carbon and nitrogen sources for producing therapeutic DNA vaccine pcDNA-CCOL2A1. **a** Evaluation of the optimal basal culture medium among LB, 2 × YT, TB, M9 (Glycerol) and M9 (Glucose) in shaking flask culture through One-Way ANOVA. **b** Screen the optimal carbon and nitrogen sources in shaking flask culture using One-Way ANOVA. *p < 0.05, * * *p* < 0.001. Data are expressed as the mean ± standard deviation (SD) of 3 independent experiments

Several previous studies have reported the precedence of single-factor analysis before using PBD and BBD [22, 23]. Accordingly, initial screening was performed for the selection of optimum basal culture medium, wherein 2 × YT was found to be advantageous for the yield of plasmid DNA vaccine pcDNA-CCOL2A1 produced by the engineered *E. coli* DH5α. Further evaluation of carbon and nitrogen sources indicated that mannitol and peptone can significantly increase plasmid yield compared with the basal culture medium 2 × YT (*p*<0.05), as shown in Fig. 1a-b.

### PBD screening elucidated the key variables affecting the yield of therapeutic DNA vaccine pcDNA-CCOL2A1

In the PBD experiment, ten variables were chosen to screen the key factors affecting the yield of plasmid DNA vaccine, as shown in Table 1. The data reported in Table 2 showed a substantial variation in plasmid yield among the 12 experimental sets, varying from 146.60 ± 15.25 mg/L to 312.86 ± 13.69 mg/L under two different levels of factors. Based on regression analysis of PBD in Table 3, the fitting model for the yield of plasmid DNA vaccine was significant (*p* = 0.0287). The ratio of adequate precision measures the signal-to-noise ratio, and a ratio greater than 4 is desirable. In this case, adequate precision was 84.831, confirming that the model could adequately navigate the design space. The goodness of the model was checked by the determination coefficient R^2^, which was 0.9999. Among these factors, peptone, yeast extract, mannitol, and inoculum concentration were the significant model terms on the response (*p<*0.05). The three most significant variables were peptone, mannitol, and inoculum concentration, and their contributions to the yield of plasmid DNA vaccine were 48.18%, 21.56%, and 21.55% respectively. In particular, these three variables exerted a positive effect on plasmid production. Other independent variables with *p*>0.05 were generally considered insignificant and would be not included in the subsequent optimizing step. Thereafter, the culture conditions were reduced to three most significant variables: peptone, mannitol, and inoculums concentration. The precise optimal values of the individual variables were still unknown but could be determined through subsequent BBD.

**Table 1:** Variables and their levels used in Plackett-Burman design for screening of culture conditions affecting the yield of plasmid DNA vaccine pcDNA-CCOL2A1 by the engineered *E. coli* DH5α

Symbol code	Factors	Experimental values	
		Low level (− 1)	High level (+1)
A(g/L)	Peptone	16.00	24.00
B(g/L)	Yeast extract	10.00	15.00
C(g/L)	NaCl	5.00	7.50
D(g/L)	Ampicilin	0.05	0.10
E(mL/L)	Microelement	10.00	15.00
F(g/L)	Mannitol	5.00	7.50
G	Initial pH	6.00	8.00
H(RPM)	Rotational speed	180	250
J(°C)	Temperature	30.00	37.00
K(OD)	Inoculum concentration	0.10	0.20
L	Dummy factors

**Table 2:** Plackett–Burman design matrix with response value for screening of culture conditions affecting the yield of plasmid DNA vaccine pcDNA-CCOL2A1 by the engineered *E. coli* DH5α

Run	Variable levels											Plasmid yield
	A	B	C	D	E	F	G	H	J	K	L	(mg/L)
1	1	−1	1	1	−1	1	1	1	− 1	−1	− 1	258.29 ± 6.44
2	−1	1	1	−1	1	1	1	− 1	− 1	− 1	1	202.98 ± 7.37
3	1	−1	− 1	− 1	1	− 1	1	1	− 1	1	1	267.36 ± 11.00
4	−1	− 1	− 1	−1	− 1	−1	− 1	−1	− 1	−1	− 1	158.51 ± 11.30
5	−1	−1	− 1	1	− 1	1	1	−1	1	1	1	234.83 ± 14.17
6	1	1	1	−1	−1	−1	1	−1	1	1	−1	279.81 ± 10.27
7	1	1	−1	1	1	1	−1	− 1	− 1	1	− 1	312.86 ± 13.69
8	−1	1	1	1	−1	− 1	−1	1	− 1	1	1	198.70 ± 11.36
9	−1	−1	1	−1	1	1	−1	1	1	1	−1	220.79 ± 8.51
10	1	1	−1	−1	− 1	1	− 1	1	1	−1	1	284.63 ± 10.09
11	−1	1	−1	1	1	−1	1	1	1	−1	− 1	146.60 ± 15.25
12	1	−1	1	1	1	−1	− 1	−1	1	−1	1	181.27 ± 10.67

Data are expressed as the mean ± standard deviation (SD) of 3 batches independent experiments for each strain

**Table 3:** The regression analysis of variance for Plackett–Burman factorial model for the yield of plasmid DNA vaccine

Code	Term	Stdized Effects	%Contribution	*F*-Value	*p*-Value
	Model			734.21	0.0287
A	Peptone(g/L)	70.30	48.18	3538.00	0.0107*
B	Yeast extract(g/L)	17.42	2.96	217.36	0.0431*
C	NaCl(g/L)	−10.49	1.07	78.81	0.0714
D	Amp(g/L)	−13.59	1.80	132.16	0.0552
E	Microelement(mL/L)	−13.82	1.86	136.70	0.0543
F	Mannitol(g/L)	47.02	21.56	1582.96	0.0160*
G	Initial pH	5.52	0.30	21.79	0.1344
H	Rotational speed(RPM)	1.02	0.010	0.74	0.5470
J	Temperature(°C)	−8.46	0.70	51.27	0.0883
K	Inoculum concentration(OD)	47.01	21.55	1582.33	0.0160*

R^2^ = 0.9999, adj-R^2 ^= 0.9985, Adequate precision = 84.831

*Identified variables with a significant effect on the response (*p*-value <0.05)

### The steepest ascent experiment optimized the key variables affecting the yield of therapeutic DNA vaccine pcDNA-CCOL2A1

Based on the analysis of the screening design, the path of steepest ascent was then applied to determine the most suitable direction for changing the variable ranges. As the three most significant variables exerted a positive effect on plasmid production, the direction of steepest ascent should increase their concentration to approach the optimal experimental region of maximum response. Five sets of experiments of the steepest ascent and corresponding experimental results were showed in Table 4. The yield of plasmid DNA vaccine peaked at the third step and no further improvement could be achieved in there sponse when peptone, mannitol, and inoculum concentration were selected to be 26 g/L, 8 g/L and 0.35, respectively, which suggested that it was proximal to the region of maximum response. Accordingly, these levels of the three factors in the third set were considered the center point of BBD.

**Table 4:** Steepest ascent experiments to move the experimental region towards the maximum yield of plasmid DNA vaccine pcDNA-CCOL2A1 by the engineered *E. coli* DH5α

Run	Peptone(g/L)	Mannitol (g/L)	Inoculum concentration (OD)	Plasmid yield (mg/L)
1	22	6	0.15	296.92 ± 10.00
2	24	7	0.25	313.76 ± 8.01
3	26	8	0.35	338.57 ± 11.92
4	28	9	0.45	326.04 ± 8.65
5	30	10	0.55	303.95 ± 8.46

Data are expressed as the mean ± standard deviation (SD) of 3 batches independent experiments for each strain

### BBD optimized the screened culture conditions for the yield of therapeutic DNA vaccine pcDNA-CCOL2A1

Preliminary trials confirmed that peptone (24–28 g/L), mannitol (7~9 g/L), and inoculum concentration (0.25~0.45) were suitable. In the present analysis, experiments were designed to obtain a second-order polynomial equation consisting of 12 trials plus 5 central points. The design matrix of the variables was showed in Table 5 along with the experimental values of response. Through multiple regression analysis of the experimental data, shown in Table 5, the following second-orderpolynomial equation was derived for the plasmid yield by only considering the significant terms:

$$ {\displaystyle \begin{array}{c}Y=338.78-3.70\times \mathrm{A}+5.06\times \mathrm{B}+3.98\times \mathrm{C}+10.52\times \mathrm{A}\times \mathrm{B}\\ {}+3.18\times \mathrm{A}\times \mathrm{C}-5.98\times \mathrm{B}\times \mathrm{C}-18.12\times {\mathrm{A}}^2-23.34\times {\mathrm{B}}^2-15.89\times {\mathrm{C}}^2\end{array}} $$

**Table 5:** Through BBD optimizing the screened culture conditions for the yield of plasmid DNA vaccine pcDNA-CCOL2A1 by the engineered *Escherichia coli* DH5α

Run	A-Peptone(g/L)		B-Mannitol(g/L)		C-Inoculum concentration(OD)		Plasmid yield (mg/L)
	Code level	Real level	Code level	Real level	Code level	Real level	
1	1	28	−1	7	0	0.35	277.80 ± 8.38
2	1	28	0	8	− 1	0.25	293.63 ± 5.63
3	1	28	0	8	1	0.45	307.20 ± 6.81
4	0	26	−1	7	1	0.45	305.73 ± 9.32
5	0	26	1	9	−1	0.25	305.33 ± 11.90
6	0	26	0	8	0	0.35	332.53 ± 14.61
7	0	26	0	8	0	0.35	335.60 ± 10.52
8	0	26	0	8	0	0.35	339.70 ± 10.91
9	0	26	0	8	0	0.35	345.10 ± 13.60
10	0	26	−1	7	−1	0.25	285.03 ± 8.23
11	−1	24	0	8	1	0.45	309.53 ± 9.69
12	−1	24	0	8	−1	0.25	308.70 ± 10.68
13	-1	24	1	9	0	0.35	295.80 ± 10.61
14	0	26	1	9	1	0.45	302.10 ± 14.45
15	1	28	1	9	0	0.35	310.73 ± 10.71
16	0	26	0	8	0	0.35	340.97 ± 13.16
17	-1	24	-1	7	0	0.35	304.93 ± 8.88

Data are expressed as the mean ± standard deviation (SD) of 3 batches independent experiments for each strain

Where Y is the predicted response of plasmid yield, A, B, and C are the coded values of peptone, mannitol, and inoculum concentration, respectively. Statistical significance of the second-order model and all the coefficient estimates were assessed using ANOVA, and the data are shown in Table 6. The quadratic regression model was highly significant, which was evident from the F-test with a very low probability value (*p*<0.0001). The value of adj-R^2^ (0.9626) suggested that the total variation of 96.26% for the yield of plasmid DNA vaccine was attributed to the independent variables. The determination coefficient (*R*^2^ = 0.9836), which is commonly used to assess the goodness of the model, exhibited an excellent correlation between the experimental and predicted response values. Alow CV (CV = 1.25%) value clearly revealed that the deviations between experimental and predicted values were low and it displayed not only a high degree of precision but also high reliability in conducted experiments. Adequate precision measures the signal-to-noise ratio, and a ratio greater than 4 is desirable. In this study,a ratio of 20.387 indicated an adequate signal. Therefore, the quadratic model was selected in this optimization study. Table 6 showed the corresponding *p*-value and the parameter estimate.

**Table 6:** Regression analysis of a full second-order polynomial model for the optimized yield of plasmid DNA vaccine by the engineered *E. coli* DH5α

Term	Sum of Squares	df	Mean Square	*F*-Value	*p*-value
Model	6344.09	9	704.90	46.72	<0.0001*
A	109.52	1	109.52	7.26	0.0309*
B	204.70	1	204.70	13.57	0.0078*
C	126.94	1	126.94	8.41	0.0230*
AB	442.39	1	442.39	29.32	0.0010*
AC	40.54	1	40.54	2.69	0.1452
BC	143.20	1	143.20	9.49	0.0178*
A^2^	1382.96	1	1382.96	91.65	<0.0001*
B^2^	2293.73	1	2293.73	152.01	<0.0001*
C^2^	1063.13	1	1063.13	70.46	<0.0001*
Residual	105.62	7	15.09		
Lack of fit	10.92	3	3.64	0.15	0.9221
Pure Error	94.71	4	23.68		
Cor Total	6449.71	16			

R^2^ = 0.9836, adj-R^2^ = 0.9626, Adequate precision = 20.387, CV = 1.25%, Adequate precision = 20.387

*Identified variables with a significant effect on the response (*p*-value<0.05)

**Fig. 2:**
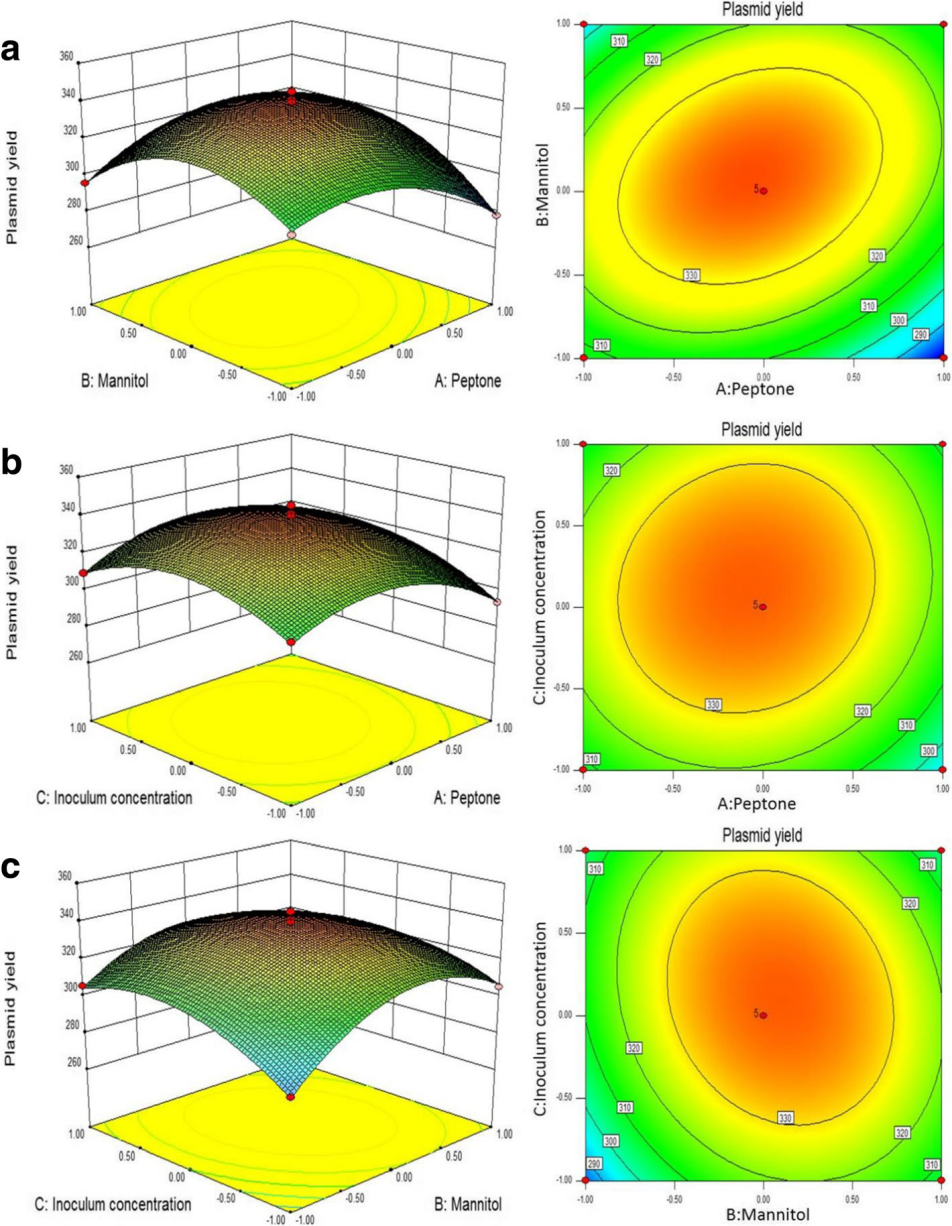
**a**, **b**, **c** Response surface 3D model (left) and contour plot (right) to assessthe effects of the three variables on the yield of plasmid DNA vaccine pcDNA-CCOL2A1 produced by the engineered *E. coli* DH5α. **a** response surface plot showing the mutual effect of peptone and mannitol on the yield of plasmid DNA vaccine pcDNA-CCOL2A1; **b** response surface plot showing the mutual effect of peptone and inoculum concentration on the yield of plasmid DNA vaccine pcDNA-CCOL2A1; **c** response surface plot showing the mutual effect of mannitol and inoculum concentration on the yield of plasmid DNA vaccine pcDNA-CCOL2A1

This multiple nonlinear model resulted in three response surface graphs through canonical analysis of the response surface. Interpretation of the response surface 3D model and contour plot were the graphical representations of regression equation. They provided visual interpretations of the relationship between responses and experimental levels of each variable, and the type of interactions between two test variables. Fig. 2a was the fitted response surface 3D model and their corresponding contour plots for the yield of plasmid DNA vaccine produced by the predicted model, respectively. Fig. 1a shows that the yield of plasmid DNA vaccine significantly increased with peptone increasing from 24 to 25.86 g/L, mannitol increasing from 7 to 8.08 g/L, but decreased beyond this centerpoint, reaching a maximum yield of 339.03 mg/L. The effect of peptone and mannitolon the yield of plasmid DNA vaccine was also sensitive within the tested range, which was proved by the *p*-value (0.0309, 0.0078) in Table 6. Furthermore, the significant interaction of peptone and mannitol could be easily explained by its elliptical shape of the contour plot and *p*-value (0.001). It was also noticed in Fig. 2b-c that the response presented downward movement when the value of variables was higher than the center point, indicating the existence of the maximum predicted value of the yield of plasmid DNA vaccine. The statistical optimal values of variables were obtained when moving along the major and minor axes of the contour and the response at the center point yields the maximum plasmid production. These observations were also verified through canonical analysis of the response surface. By solving the inverse matrix from the second-order polynomial equation, the optimum values of the test variables were peptone, 25.86 g/L;mannitol, 8.08 g/L; inoculum concentration, 0.36. Under the optimal conditions, the maximum predicted the yield of plasmid DNA vaccine was 339.32 mg/L. To confirm the validity of the model for predicting the maximum yield of plasmid DNA vaccine, an additional experiment using this optimum operation conditions was performed under shake-flask culture. The average yield of plasmid DNA vaccine was 341.86 ± 10.67 mg/L (*N* = 3). The results were closely related to the data obtained from optimization analysis, suggesting that the RSM model was adequate for reflecting the expected optimization, and the model was satisfactory and accurate.

## Discussion

The final acquisition of plasmid DNA vaccines with the highest yield, purity, and quality were closely related to not only the upstream antigen-specific genes for disease targets, the most appropriate expression vectors, and the appropriate *Escherichia coli* strains for production, but also the optimized fermentation conditions, culture media, and scale-up as well as the downstream purification technology [24–30]. In the present study, we have optimized the fermentation conditions at a shake-flask level for the engineered *Escherichia coli* DH5α to for high yield of therapeutic DNA vaccine pcDNA-CCOL2A1 through combined PBD with BBD-RSM, by which the yield of therapeutic DNA vaccine pcDNA-CCOL2A1 was markedly increased.

In practice terms, the medium compositions such as the basal culture media, the carbon sources, the nitrogen sources, the carbon/nitrogen ratio (C/N), amino acid starvation, etc., as the essential factors for the fermentation conditionsfor the production of plasmid DNA vaccines are usually the first to be chosen and optimized in the beginning of the fermentation condition optimization for increasing plasmid DNA production in *E. coli* strains [14, 15]. The fermentation condition optimization, including screening of optimal medium compositions, is influenced by many factors, among which, interactions may exist. The routine single-dimensional studies changing one independent variable at a time and maintaining the others constant yields unreliable results, inaccurate conclusions, and even frequent interactions of two or more factors [17]. Thus, it is necessary to apply reasonable experimental designs and optimization methodologies in condition screening and process optimization. Because *E. coli* strain DH5a used in the present study was selected typically for plasmid DNA production [31], we first used single-factor analysis to evaluate several basal culture media commonly used for culturing DH5a, which include LB, 2 × YT, TB, M9 (Glycerol) and M9 (Glucose). Finally, we screened 2 × YT as the optimal basal culture medium, mannitol as the optimal carbon source, and peptone as the optimal nitrogen source. In theory, the production of plasmid DNA vaccines is also affected by varying both carbon and nitrogen concentrations [30, 32–34]. Thus, we applied PBD to further screen out the three most significant factors affecting the yield of therapeutic DNA vaccine pcDNA-CCOL2A1, followed by the paths of steepest ascent to move to the nearest region of maximum response. The most significant factors identified through PBD were peptone, mannitol, and inoculum concentrations. Together, our results indicate that PBD is efficient in screening medium components at the shake-flask level and has been widely used in the optimization of fermentation conditions [18, 19]. This technique cannot determine the exact quantity but can provide indication and tendency regarding the necessity of each variables in relatively few experiments.

In the present study, we used RSM to further optimize the yield of therapeutic DNA vaccine pcDNA-CCOL2A1 by BBD. RSM not only helped locate the optimum levels of the most significant factors but also proved to be useful and satisfactory in this process-optimizing practice. Through these optimization experiments, the maximum yield of plasmid DNA vaccine at 339.32 mg/L was obtained under the optimum conditions with peptone (25.86 g/L), mannitol (8.08 g/L), and inoculum concentration (OD = 0.36), which is significantly higher than those of most studies. Most current fermentation media and processes have only resulted in low yields of plasmid DNA (< 200 mg/L) [14, 35], though a few have resulted in high yields (500–1500 mg/L) [36–38]. Compared with the original medium, an increase of 51.9% was obtained. The predicted plasmid yield was closely related with the experimental value, which was 341.86 ± 10.67 mg/L (*N* = 3). Further studies are required to assess the optimization of fermentation conditions involving in several major factors such as growth conditions, culture types, culture medium compositions, etc. In the present study, we obtained a higher yield of plasmid DNA vaccine by only optimizing the two factors of the components of the nutrient medium and inoculum concentration. Hence, further optimization of fermentation conditions including growth conditions and culture types would significantly increase both the yield and productivity of therapeutic DNA vaccine pcDNA-CCOL2A1. These optimization methods for fermentation conditions are currently being investigated in our laboratory.

## Conclusions

In summary, the fermentation medium and conditions of the engineered *Escherichia coli* DH5α producing a novel therapeutic DNA vaccine pcDNA-CCOL2A1 were scientifically selected and optimized by RSM. Under the optimum conditions with peptone (25.86 g/L), mannitol (8.08 g/L), and inoculum concentration (OD = 0.36), the maximum yield of plasmid DNA vaccine at 339.32 mg/L was obtained, with an increase of 51.9%. In addition to this, we conducted experiments under the optimal conditions.The experimental value was 341.86 ± 10.67 mg/L (*N* = 3), which was closely related with the predicted plasmid yield. The present results will provide a robust foundation for further pilot-scale tests and industrial-scale production of final high-quality and high-yield therapeutic DNA vaccine pcDNA-CCOL2A1 for RA in the near future.

## Methods

### Plasmid and bacterial strains

Eukaryotic expression vector for producing therapeutic DNA vaccine pcDNA-CCOL2A1 was previously constructed in our laboratory, which contains a 4837 bp cDNA sequence encoding the chicken type II procollagen gene, but lacking the N-propeptides. To obtain high levels of CCOL2A1 gene expression, both the signal sequence and the Kozak consensus sequence were inserted into pcDNA™3.1(+), a highly stable vector used for transient gene expression [9, 39]. The resulting recombinant plasmid containing an ampicillin resistance gene for selection was cloned in *E. coli* DH5α (CB101; Tiangen, Beijing, China).

### Medium and cultivation

Media included Luria-Bertani (LB) [10 g/L tryptone, 5 g/L yeast extract, 10 g/L NaCl, pH 7.0], Tartof and Hobbs (TB)[12 g/L tryptone, 24 g/L yeast extract, 0.4% glycerol, 2.31 g/L KH_2_PO_4_, 12.54 g/L K_2_HPO_4_], M9 [0.01 g/L CaCl_2_, 0.24 g/L MgSO_4,_ 12.8 g/L Na_2_HPO_4_·7H_2_O, 3 g/L KH_2_PO_4_, 0.5 g/L NaCl, 1 g/L NH_4_Cl, 20% Glucose or Glycerol], 2 × YT [16 g/L tryptone, 10 g/L yeast extract, 5 g/L NaCl, pH 7.0], and Microelement mix [0.01 g/L MnSO_4_·7H_2_O, 0.05 g/L ZnSO_4_·7H_2_O, 0.01 g/L H_3_BO_3_, 0.01 g/L CaCl_2_·2H_2_O, 0.01 g/L Na_2_MoO_4_, 0.2 g/L CoCl_2_·6H_2_O, 0.01 g/L AlK(SO_4_)_2_·12H_2_O, 0.001 g/L NiCl_2_·6H_2_O] [40]. Engineered *Escherichia coli* DH5α were cultured in 100 mL of medium in 500 mL Erlenmeyer flasks with the initial inoculum concentration of OD_600_ = 0.1, and incubated at 37 °C on a rotary shaker at 220 rpm. After 16 h of incubation, plasmid DNA was purified from the bacterial cell, using the Wizard^R^ Plus SV Minipreps DNA Purification System (Promega, USA). The concentrations of the plasmid pcDNA-CCOL2A1 were measured at OD_260_ and OD_280_ using Synergy™ HT Multi-Mode Microplate Reader (BioTek Instruments, Inc., Winooski, VT, USA).

### Single-factor analysis

In each experiment, one factor was changed with the other factors remaining constant. The initial evaluation was performed to identify the optimal basal culture medium from LB, 2 × YT, TB, M9 (glycerol) and M9 (glucose). The effect of various carbon and nitrogen sources was also determined through single-factor analysis. Carbon sources (5 g/L glycerol, glucose, and mannitol) were evaluated, while other components were maintained constant as basal culture medium. The nitrogen sources (5 g/L peptone, NH_4_Cl, urea) were analyzed with other constituents as that of basal culture medium. Although this method is time-consuming, it is propitious to the selection of level in PBD, rendering the results more reasonable and credible.

### Plackett–Burman design for screening

Multiple regression analysis and analysis of variance (ANOVA) were conducted for fitting the mathematical model using Design Expert software (Version 8.0.6, Stat-Ease Inc., Minneapolis, MN, USA). Ten variables (peptone, yeast extract, NaCl, Ampicillin [Amp], microelements, mannitol, rotational speed, pH, fermentation temperature, and inoculum concentration) were assessed using PBD and the model was evaluated using the F-test and goodness of fit through multiple correlations R. Each independent variable was tested at two levels, high and low, which are denoted by (+) and (−), respectively. The experimental design with the name, symbol code, and actual levels of the variables are shown in Tables 1 and 2 shows details of the design matrix. In this study, 12 experiments were conducted and the most optimal variables were selected for further evaluation. Based on regression analysis of the variables, significant levels at 95% level (*p<*0.05) were considered to significantly affect the yield of the plasmid vaccine.

### Path of the steepest ascent experiment

After having identified the three most significant variables through the PBD, the steepest ascent experiment was performed to move the experimental region of the response in the direction of the optimum, by appropriately changing the range of the selected variables. The path initiated from the design center of the factorial design (the screening design) and receded when no further improvement in the response could be achieved. When the maximum value was gained, that point could be considered as the center point for the optimization experimental design [31]. Table 4 summarizes the experimental design, the variables, and their values.

### Box-Behnken design

The RSM is a collection of statistical tools and techniques for constructing and exploring a putative functional relationship between a response variable (i.e., plasmid yield) and a set of design variables (i.e., peptone, mannitol, and inoculum concentration). It is possible to derive an expression for performance measurement on the basis of the response values obtained from experiments using a particular combination of input variables [41]. In the present study, by employing BBD and RSM, the effects of the three independent variables (peptone, 24–28 g/L; mannitol, 7–9 g/L; inoculum concentration, OD = 0.25–0.45) and three levels (high, middle, and low) on the response (plasmid yield) were investigated to determine the optimal conditions, which maximized the yield of therapeutic DNA vaccine pcDNA-CCOL2A1 from shake cultivation. Each independent variable was coded at three levels: − 1, 0, and + 1. The BBD comprised 17 experiments with five center points (to allow for estimation of pure error) and facilitated calculations of response function at intermediate levels, fitting a second-order response surface. Table 4 shows the variables and their values and the experimental design. This methodology allows for modeling of a second-order equation that describes the process. Plasmid production was analyzed through multiple regression analysis through the least squares method to fit the following equation:

$$ \mathrm{Y}={\upbeta}_0+\sum {\upbeta}_{\mathrm{i}}{\mathrm{x}}_{\mathrm{i}}+\sum {\upbeta}_{\mathrm{i}\mathrm{j}}{\mathrm{x}}_{\mathrm{i}}{\mathrm{x}}_{\mathrm{j}}+\sum {\upbeta}_{\mathrm{i}\mathrm{i}}{{\mathrm{x}}_{\mathrm{i}}}^2 $$

Where Y is the measured response variable; β_0_, β_i_, β_ij_, and β_ii_ are constants and regression coefficients of the model, and x_i_ and x_j_ represent the independent variables in coded values. Data from the BBD for the optimization of plasmid production was subjected to second-order multiple regression analysis using the least squares method to obtain the parameter estimators of the mathematical model [18, 42]. Second-order multiple regression analysis was performed using the Design Expert software (Version 8.0.6, State-Ease Inc., Minneapolis, MN, USA) statistical package. The model was further assessed using ANOVA.

## Abbreviations

BBD: Box-Behnken design
BBD-RSM: Box-Behnken design-response surface method
CCOL2A1: Chicken type II procollagen
CIA: Collagen-induced arthritis
DMARDs: Disease-modifying antirheumatic drugs
MTX: Methotrexate
PBD: Plackett-Burman design
RA: Rheumatoid arthritis
RSM: Response surface method
